# Effect of Using Triclosan-Impregnated Polyglactin Suture to Prevent Infection of Saphenectomy Wounds in CABG: A Prospective, Double-Blind, Randomized Clinical Trial

**DOI:** 10.21470/1678-9741-2019-0048

**Published:** 2019

**Authors:** Paulo Samuel Santos Filho, Marisa Santos, Alexandre Siciliano Colafranceschi, Andrea Nunes de Souza Pragana, Marcelo Goulart Correia, Heloisa Helena Simões, Fernando Alves Rocha, Maria Eduarda de Vasconcelos Soggia, Ana Paula Malta Samuel Santos, Annie de Azeredo Coutinho, Matheus Swarovsky Figueira, Bernardo Rangel Tura

**Affiliations:** 1 Department of Cardiovascular Surgery, Instituto Nacional de Cardiologia (INC), Rio de Janeiro, RJ, Brazil.; 2 Núcleo de Avaliação de Tecnologia em Saúde (NATS), Instituto Nacional de Cardiologia (INC), Rio de Janeiro, RJ, Brazil.; 3 Department of Biostatistics and Bioinformatics, Instituto Nacional de Cardiologia (INC), Rio de Janeiro, RJ, Brazil.

**Keywords:** Wound Infection, Sutures, Triclosan, Coronary Artery Bypass Graft, Cardiopulmonary Bypass, Erythema, Body Mass Index, Analgesics, Pain

## Abstract

**Objective:**

To evaluate the efficacy of triclosan-coated suture for the reduction of infection in saphenectomy wounds of patients undergoing coronary artery bypass graft (CABG) surgery.

**Methods:**

A total of 508 patients who underwent saphenectomy in CABG surgery were included in a prospective, randomized, double-blind trial from February/2011 to June/2014. Patients were randomized into the triclosan-coated suture group (n= 251) and the conventional non-antibiotic suture group (n=257). Demographic (gender and age), clinical (body mass index, diabetes, and use of analgesics), and intraoperative (cardiopulmonary bypass and cross-clamp times) variables and those related to the saphenectomy wound (pain, dehiscence, erythema, infection, necrosis, and hyperthermia) were measured and analyzed.

**Results:**

Of the 508 patients who underwent saphenectomy, 69.9% were males and 40.2% were diabetic. Thirty-three (6.5%) patients presented infection: 13 (5.3%) with triclosan and 20 (7.9%) with conventional suture (*P*=0.281). Among diabetic patients (n=204), triclosan suture was used in 45.1% with four cases of infection; conventional suture was used in 54.9% of them, with 11 cases of infection. Most patients (94.3%) underwent on-pump CABG. Wound pain was observed in 9.9% of patients with triclosan-coated suture and in 17.9% with conventional suture (*P*=0.011). Wound hyperthermia was found in 1.6% of patients with triclosan-coated suture and in 5.4% of those with conventional suture (*P*=0.028).

**Conclusion:**

Triclosan-coated suture shows lower infection rate in saphenectomy of patients undergoing CABG, although the differences were not statistically significant. Pain and wound hyperthermia were less frequent in patients with triclosan-coated sutures compared with conventional sutures.

**Table:** 

Abbreviations, acronyms & symbols	
A	= Absence
BMI	= Body mass index
CABG	= Coronary artery bypass graft
CONSORT	= Consolidated Standards of Reporting Trials
CPB	= Cardiopulmonary bypass
INC	= Instituto Nacional de Cardiologia
P	= Presence
SIRS	= Systemic inflammatory response syndrome
SSI	= Surgical site infection
SUS	= Sistema Único de Saúde

## INTRODUCTION

Cardiovascular disease is the world's leading cause of mortality, morbidity, and disability^[1,2]^. In several clinical conditions, the coronary artery bypass graft (CABG) surgery has been effective in relieving these patients’ symptoms and increasing survival rates^[3]^. However, such surgery requires highly specialized hospitals and trained staff, generating high costs for health systems. CABG is the most practiced cardiac surgery in our country, most of which is performed by the Sistema Único de Saúde (SUS), the Brazilian public health system, both in public and in philanthropic or private hospitals^[4]^. Although CABG is the most appropriate treatment for coronary artery disease, currently, the major challenge is to reduce postoperative intercurrences, such as wound infections, dehiscence, and pain, especially in the saphenectomy, presenting complications in 10% of the cases, which can cause greater morbidity and time and cost of hospitalization^[5]^. Although there has been an increase in the use of arterial conduits for grafting in CABG surgery, the saphenectomy, a technique used to obtain venous graft during operative intervention, is one of the most performed procedures in the world^[6,7]^ and it remains essential for obtaining the goal of complete myocardial revascularization in most patients^[8]^. Complications related to the surgical technique such as hematoma, seroma, suture dehiscence, incision border necrosis, lymphedema, pain, or infection occur in up to 30% of patients undergoing saphenectomy^[6,9,10]^. Surgical site infection (SSI) after CABG represents a high morbidity rate, which impedes continuity of treatment and increases the length of hospital stay and, consequently, the medical and hospital expenses^[11-15]^. Several risk factors for saphenectomy infection were identified, including obesity, age over 75 years, female gender, diabetes, previous stroke, and postoperative transfusion of five units or more of red blood cells^[14]^. Another factor that may influence the incidence of SSI in patients undergoing CABG is the type of suture used in the surgical wound closure, since bacteria can adhere to the suture material^[16]^. Sutures can be coated with antibacterial substances that may reduce microbial load on the wound. Triclosan (2,4,4'-trichloro-2'-hydroxydiphenyl ether) is an antibacterial substance which, in preclinical studies, has reduced bacterial growth through the inhibition of fatty acid synthesis^[17]^. Triclosan-coated sutures have been tested in patients undergoing saphenectomy during CABG with divergent results^[18,19]^.

In Brazil, despite the high number of CABG surgeries performed^[4]^, studies on infection in the saphenectomy after CABG have not been found in the literature. In addition, it is important to note that in this study all cardiac surgeries were performed by the same team and in a teaching hospital. The aim of this study was to assess the efficacy of triclosan-coated suture in reducing infection in the saphenectomy wounds of CABG patients.

## METHODS

A prospective, randomized, double-blind clinical trial studied 508 patients undergoing saphenectomy during CABG, with and without cardiopulmonary bypass (CPB), regardless of race. The patients were attended at the Cardiovascular Surgery Service of the Instituto Nacional de Cardiologia (INC), Ministry of Health, Rio de Janeiro/RJ, Brazil, between February/2011 and June/2014. This study was approved by the INC’s Research Ethics Committee (Protocol No. 0281/21.05.2010) and was registered on the Registro Brasileiro de Ensaios Clínicos - ReBEC - number RBR-4gfk87.

Patients who underwent consecutively, prospectively, and exclusively on-pump and off-pump CABG, of both genders, and aged >30 years met the inclusion criteria for the study. Patients undergoing CABG associated with other cardiac surgeries (valvar surgeries, ventricular aneurysms, acquired ventricular septal defects, congenital heart diseases) or undergoing vascular surgeries other than CABG; bilateral saphenectomized patients; pregnant women; patients under antibiotic therapy for previous infectious disease up to a month before; immunosuppressed patients (acquired immune deficiency syndrome, neoplasia, and or use of corticosteroids > 0.5 mg/kg/day); patients requiring simultaneous carotid artery surgery; and patients with severe peripheral vascular disease, history of venous disease of the deep system and superficial thrombophlebitis of the great saphenous vein, and with psychiatric disorder were excluded from the study.

The patients were divided into two groups according to the type of suture used to close the saphenectomy wounds: triclosan-coated 910 polyglactin suture (Vicryl® Plus) (n=251) and conventional 910 polyglactin suture (Vicryl®) (n=257), both from the same manufacturer.

We performed preoperative decolonization with a chlorhexidine bath one hour before going to the surgical center and using nasal Mupirocine twice a day during the five days before surgery. Trichotomy was done with electric tricotomizer only in the incision site, in the operating room, and asepsis was done with soap chlorhexidine followed by alcoholic chlorhexidine. The surgical incision was made between 30 and 60 minutes after the infusion of antibiotic prophylaxis according to the routine of the INC’s Comissão de Controle de Infecção Hospitalar, the hospital’s infection control commission. We closed all saphenectomies with Vicryl and Vicryl Plus sutures, in three levels in the thighs and in two levels in the legs.

At randomization, a table was generated using a specific computational routine. The cardiovascular surgeon did not have prior access to the table (allocation was concealed). A blocked randomization scheme was used, with block sizes of 2, 4, or 6. This table remained blinded to all participants in the surgical procedure, as well as to all those who were involved in its follow-up, except for the professionals responsible for randomization and masking. In the masking process, counselors, the nurses responsible for the randomization, the secretary, and surgical technologists learned about the drawn sutures/patients. Surgeons, the researchers and their assistants, and the patients were masked.

Patient data were obtained from control charts, including demographic (gender and age), clinical (body mass index, diabetes, and analgesic use), and intraoperative (CPB and cross-clamp times) variables and those related to the saphenectomy wounds (pain, dehiscence, erythema, infection, necrosis, and hyperthermia).

**Fig. 1:**
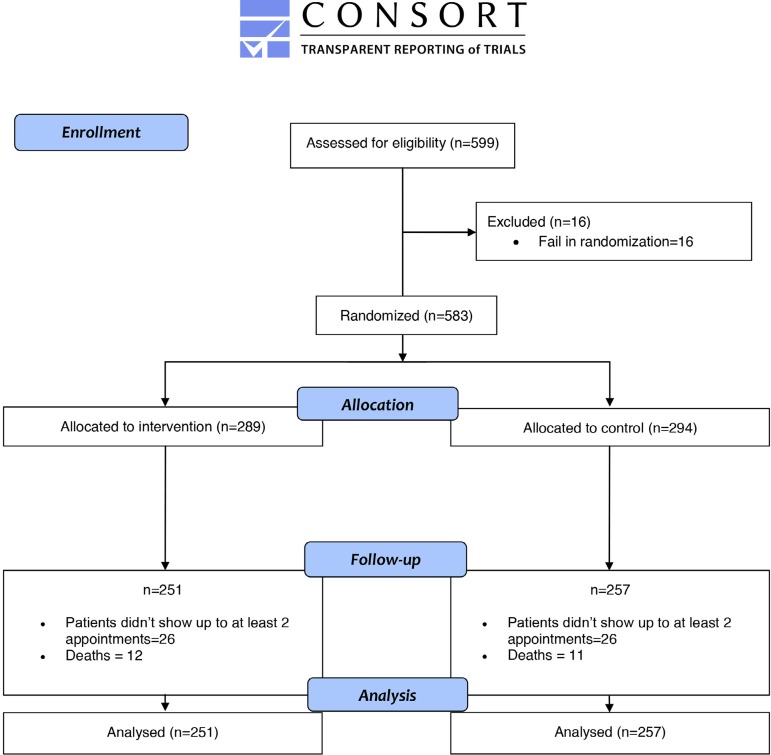
CONSORT flowchart shows the total amount of included and excluded patients, and how many sat in each phase of the essay. CONSORT=Consolidated Standards of Reporting Trials

Figure 1 shows that in the follow-up 38 patients were excluded from the intervention group (26 patients didn’t show up to at least two appointments and 12 died) and 37 patients were excluded from the control group (26 patients didn’t show up to at least two appointments and 11 died).

The sutures were evaluated and photographed around the 7^th^, 14^th^, and 30^th^ day of saphenectomy. Saphenectomy wound infection was defined as hyperemia and peri-border cellulitis with opening (dehiscence or necrosis) of 3 cm or more in the longitudinal direction and drainage of purulent secretion within 30 days of the surgical procedure. Wound pain and hyperthermia were evaluated three times during 30 days after saphenectomy considering the scale 0 = absence, 1 = mild, 2 = moderate, and 3 = severe for pain, and cold, normal, and warm for temperature.

### Statistical Analysis

Descriptive statistics were used to characterize the sample. Continuous variables were expressed as mean ± standard deviation and compared using unpaired Student’s *t*-test. Categorical variables were expressed as absolute and relative frequency (%) and compared using the Chi-square test. Differences in the distribution of numerical variables between groups were analyzed by Student’s *t*-test and Mann-Whitney U test. The differences between categorical variables were analyzed using Chi-square and Fisher's exact tests. All analyses were performed using the R software (*The R Foundation for Statistical Computing*, Vienna, Austria), version 3.3.3^[20]^. The level of significance was *P* <0.05.

## RESULTS

Of the 508 patients studied, 69.9% were males and 30.1% were females, with a mean age of 61.1 ± 9.3 years (range 36-84 years). Results of clinical variables related to saphenectomy and intraoperative wounds are described in Table 1. The most frequent findings included erythema (26.5%) and necrosis (23.6%).

**Table 1:** Clinical profile of 508 patients undergoing saphenectomy during CABG.

Variables		N (%)
Diabetes mellitus		204 (40.2)
BMI	Malnutrition	2 (0.4)
	Eutrophic	173 (36.6)
	Borderline	163 (34.5)
	Obesity	135 (28.5)
Wound pain		71 (13.9)
Dehiscence		117 (22.9)
Erythema		133 (26.5)
Infection		33 (6.6)
Gender	Female	153 (30.1)
	Male	355 (69.9)
Necrosis		118 (23.6)
Wound hyperthermia		18 (3.5)

BMI=body mass index; CABG=coronary artery bypass graft

Results of comparative analysis between groups of patients undergoing saphenectomy with triclosan-coated suture and conventional suture are shown in Table 2. There were differences between the groups for the variables pain (*P*=0.011) and wound hyperthermia (*P*=0.028), which were significantly more frequent in the conventional group than in the triclosan-coated suture group.

**Table 2:** Results of statistical analysis of groups of patients undergoing saphenectomy with triclosan-coated suture (n = 251) and conventional suture (n = 257).

Variables		Triclosan	Conventional	*P*-value
Gender	Female	76 (30.3)	77 (30.0)	1.000
	Male	175 (69.7)	180 (70.0)	
BMI (kg/m^2^)	< 18	1 (0.4)	1 (0.4)	0.866
	18 - 25	88 (38.3)	84 (34.9)	
	26 - 30	78 (33.9)	85 (35.3)	
	> 30	63 (27.4)	71 (29.5)	
Diabetes	P	92 (36.7)	112 (43.6)	0.124
	A	159 (63.3)	145 (56.4)	
Wound pain	P	25 (10.0)	46 (17.9)	0.011
	A	226 (90.0)	211 (82.1)	
Wound hyperthermia	P	4 (1.6)	14 (5.4)	0.028
	A	247 (98.4)	243 (94.6)	
Infection	P	13 (5.3)	20 (7.9)	0.281
	A	238 (94.7)	237 (92.1)	
CPB	P	238 (94.8)	241 (93.8)	0.703
	A	13 (5.2)	16 (6.2)	
Age (years)		62.01 (8.62)	60.39 (9.03)	0.024
		[60.91-63.11]	[58.95-61.35]	
		[38-83]	[36-84]	
Times (min)	CPB	87 [75.0-105.0]	90 [77.5-110.0]	0.187
	Cross-clamp	75.00 [60.3-91.0]	76 [65.0-98.5]	0.158

Mean ± standard deviation, median and variance, or number (%) A=absence; BMI=body mass index; CPB=cardiopulmonary bypass; P=presence

**Fig. 2:**
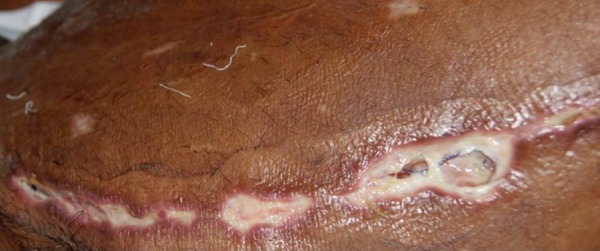
Photograph showing infection in a patient undergoing saphenectomy with Vicryl suture during coronary artery bypass graft.

**Fig. 3:**
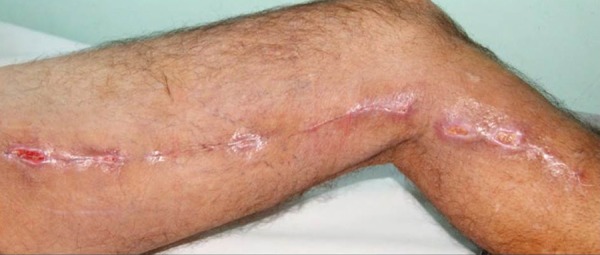
Photograph showing infection in a patient undergoing saphenectomy with Vicryl Plus suture.

**Fig. 4:**
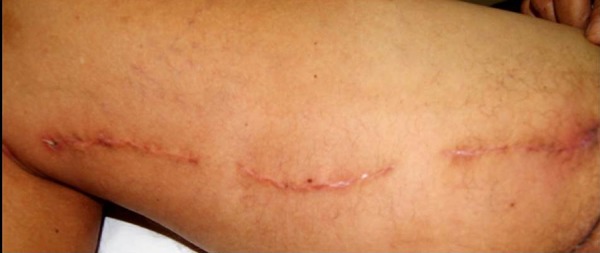
Absence of infection in a patient undergoing saphenectomy with Vicryl suture.

**Fig. 5:**
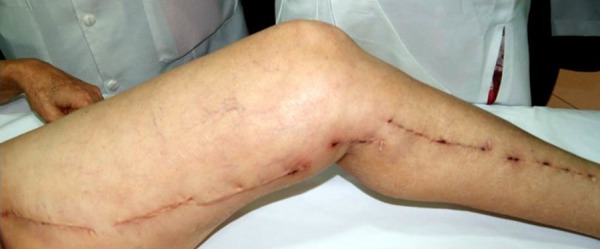
Absence of infection in a patient with Vicryl Plus suture.

Among the patients undergoing saphenectomy, 33 (6.5%) presented infection (Figures 2 and 3), being 13 (39.4%) in the triclosan group and 20 (60.6%) in the conventional group (*P*=0.281) (Table 3). The Figures 4 and 5 show saphenectomy sutures without infection. Among diabetic patients (n=204), triclosan was used in 45.1% of them, with four cases of infection, and conventional suture was used in 54.9%, with 11 cases of infection (*P*=0.284). There was a significant difference for the wound pain variable, which was observed in 9.9% of patients sutured with triclosan and in 17.9% with conventional suture (*P*=0.009)^[21]^. Wound hyperthermia was recorded in 50% of the patients with infection in the conventional group and in 15.4% of those in the triclosan-coated suture group (*P*=0.067).

**Table 3:** Results of statistical analysis of groups of patients with infection (n=33) undergoing saphenectomy with triclosan-coated suture and conventional suture.

Variables		Triclosan (n = 13)	Conventional (n = 20)	*P*-value
Gender	Female	7 (53.8)	8 (40.0)	0.4930
	Male	6 (46.2)	12 (60.0)	
BMI (kg/m^2^)	< 18	0 (0.0)	0 (0.0)	0.805
	18 - 25	4 (33.3)	7 (38.9)	
	26 - 30	5 (41.7)	5 (27.8)	
	> 30	3 (25.0)	6 (33.3)	
Diabetes	P	4 (30.8)	11 (55.0)	0.284
	A	9 (69.2)	9 (45.0)	
Wound pain	P	5 (38.5)	17 (85.0)	0.009
	A	8 (61.5)	3 (15.0)	
Wound hyperthermia	P	2 (15.4)	10 (50.0)	0.067
	A	11 (84.6)	10 (50.0)	
Age (years)		62.69 (10.80)	60.40 (7.98)	0.488
		[56.79-68.59]	[56.90-63.90]	
		[44-78]	[47-73]	

Mean ± standard deviation, median and variance, or number (%) A=absence; BMI=body mass index; P=presence

The majority of the patients (94.3%) underwent saphenectomy during on-pump CABG. The analysis of CPB and cross-clamp times showed no significant difference between the triclosan and conventional groups (Table 4).

**Table 4:** Results of statistical analysis of cardiopulmonary bypass (CPB) and cross-clamp times of groups of patients with infection (n=33) undergoing saphenectomy with triclosan-coated suture and conventional suture.

Patients		CPB time (min)	P-value	Cross-clamp time (min)	*P*-value
Triclosan	Infected	105 [90.8-136.3]	0.0774	97.5 [74.8-126.3]	0.0544
	Non-infected	88.0 [77.0-110.0]		76.0 [65.0-97.5]	
Conventional	Infected	85.0 [73.0-104.3]	0.959	69.5 [57.5-80.3]	0.342
	Non-infected	86.0 [73.0-104.8]		75.0 [60.0-91.8]	

## DISCUSSION

The results of this study show that the triclosan-coated suture presented the lowest rate of infection in saphenectomy of patients undergoing CABG. Pain and hyperthermia in the saphenectomy wound were less frequent in patients sutured with triclosan compared to those sutured with conventional suture.

In this study, considering 508 patients, the saphenectomy infection rate was 6.5%. This result is in agreement with previous studies, whose infection rates vary from 2 to 20%^[22]^. Triclosan-coated sutures have been tested on saphenectomy with controversial results. Thimour-Bergstrom et al.^[18]^, investigating triclosan-coated or non-coated sutures to close the saphenectomy wounds of 374 CABG patients, found out that there was a reduction in the infection rate in patients with triclosan sutures in 30-day (clinical visit) and 60-day (telephone interview) follow-ups. In a prospective randomized study about triclosan use in saphenectomy infection with 323 patients undergoing CABG, Seim et al.^[19]^ did not find beneficial effects of this antibacterial in the prevention of infection when compared to the conventional suture in a 30-day follow-up.

By analyzing the infection rate of the two interventions, we observed a reduction in the risk of infection of 33.4% (5.3% *vs*. 7.9% in the triclosan and conventional groups, respectively) in the saphenectomy. This risk reduction cannot be explained by any clinical differences between the two groups and taking into account the results of other articles, we should consider that it is associated with the use of triclosan-impregnated suture. Although it did not reach statistically significance, probably because the infection rate in saphenectomy was lower than expected, the result has clinical value because the use of this suture would avoid infection in every 39 patients.

Regarding variables related to saphenectomy wounds, there was a significant difference between the groups for pain and wound hyperthermia, which were less frequent in the triclosan group. However, in the comparison between patients with infection undergoing saphenectomy with triclosan-coated suture and conventional suture, only pain was statistically significant, being less frequent with triclosan suture. This result may be due to the reduced casuistic size.

Most patients underwent saphenectomy during on-pump CABG. The analysis of CPB and cross-clamp times in patients with and without infection showed no significant difference between the triclosan and conventional groups. According to Santos et al.^[23]^, the relative possibility of death in CABG surgery is 209% higher when the CPB time is greater than 115 minutes. In this study, mean CPB time was 108.6 minutes. One of the most serious problems related to CPB is the systemic inflammatory response syndrome (SIRS), characterized by clinical alterations in ventricular, pulmonary, and renal functions, coagulation disorders, susceptibility to infections, altered vascular permeability and accumulation of fluid in the interstitium, leukocytosis, vasoconstriction, and hemolysis^[24]^. Despite these changes, the body’s ability to reverse this condition and the use of corticosteroids may reduce the morbidity and mortality rates^[22]^.

Researchers have analyzed predictors of infection in patients undergoing CABG. Risk factors identified include aging, female gender, obesity, diabetes, and preoperative levels of hemoglobin^[14,19,25]^. In this study, diabetes was registered in 40.2% of the patients. However, a significant association between diabetes and infection in both groups was not found.

Although a systematic review with recent meta-analysis has found in 21 clinical trials moderate quality of evidence that triclosan-coated sutures are effective in reducing SSI^[26]^, it should be noted that infection in saphenectomy wounds in patients undergoing on-pump and off-pump CABG requires more scientific research.

The reduction of infection in the saphenectomy after CABG is fundamental for both the patient (discomfort and pain) and the hospital institution (increased hospitalization time and costs, more procedures and medication use), since this knowledge can subsidize interventions aiming at the planning and execution of new preventive strategies, minimizing the complications associated with this surgery and reducing hospital costs. This information can also be used as an indicator of quality of care in the postoperative period, in this case provided by SUS. In addition, studies on sutures coated with antibacterial substances compared to uncoated sutures in reducing SSI may contribute to the development of guidelines for the prevention of such infections^[27]^.

## CONCLUSION

In patients undergoing saphenectomy during CABG, the most frequent variables, such as demographic, clinical, and those related to saphenectomy wounds, were male gender, diabetes, erythema, and necrosis. Pain and hyperthermia in the wound were less frequent in patients sutured with triclosan. The patients with triclosan-coated sutures presented smaller infection rate in saphenectomy than those with non-coated sutures undergoing CABG, although the differences were not statistically significant.

**Table:** 

Author's roles & responsibilities	
PSSF	Substantial contributions to the conception or design of the work; or the acquisition, analysis, or interpretation of data for the work; final approval of the version to be published
MS	Substantial contributions to the conception or design of the work; interpretation of data for the work; final approval of the version to be published
ASC	Substantial contributions to the conception of the work; interpretation of data for the work; final approval of the version to be published
ANSP	The acquisition, analysis, or interpretation of data for the work; final approval of the version to be published
MGC	Collaborated in the article performing the Statistical Analysis and participating in its writing; final approval of the version to be published
HHS	The acquisition of data for the work; final approval of the version to be published
FAR	The acquisition, analysis of data for the work; final approval of the version to be published
MEVS	The acquisition, analysis of data for the work; final approval of the version to be published
APMSS	The acquisition of data for the work; final approval of the version to be published
AAC	The acquisition, analysis of data for the work; final approval of the version to be published
MSF	The acquisition, analysis of data for the work; final approval of the version to be published
BRT	Substantial contributions to the conception or design of the work; or the acquisition, analysis, or interpretation of data for the work; final approval of the version to be published
